# Influence of Hydrocolloids on the Cooking Quality and Techno‐Functional Properties of Unripe Banana Flour Pasta

**DOI:** 10.1002/fsn3.70099

**Published:** 2025-04-27

**Authors:** Siphosethu R. Dibakoane, Victor Mlambo, Belinda Meiring, July Johannes Sibanyoni, Tonna A. Anyasi, Obiro Cuthbert Wokadala

**Affiliations:** ^1^ School of Agricultural and Natural Sciences, University of Mpumalanga Nelspruit South Africa; ^2^ Tshwane University of Technology Department of Biotechnology and Food Technology Pretoria South Africa; ^3^ School of Hospitality and Tourism Management, University of Mpumalanga Nelspruit South Africa; ^4^ Food and Markets Department Natural Resources Institute, University of Greenwich Kent UK; ^5^ Medway Food Innovation Centre, Natural Resources Institute, University of Greenwich Kent UK

**Keywords:** banana flour pasta, cooking quality, hydrocolloids, techno‐functional properties, unripe banana flour

## Abstract

Banana flour is a promising ingredient for the development of functional foods due to its high resistant starch content and its gluten‐free (GF) status. However, the absence of gluten in banana flour limits its functional role in banana flour‐infused products such as pasta. This work determined the influence of three hydrocolloids including egg white (EW), guar gum (GG), and xanthan gum (XG) on the cooking parameters (cooking time and loss), color, and texture (adhesiveness and hardness) of GF unripe banana flour pasta. The pasta samples were prepared using unripe banana flour (36%) and varying levels of EW (18%–22%), GG (0.5%–4.5%), and XG (0.5%–4.5%). It was observed that there was an increase (*p* < 0.05) in the cooking time (18.67–31 min, EW; 17.33–32 min, GG), hardness (4373.99 g–5394.13 g, EW), lightness (36.3–37.9, EW), and hue (58.2–59.9, XG) of the pasta in response to incremental levels of the individual hydrocolloids. The cooking loss was highest (*p* < 0.05) at 7.9% for XG (0.5%) and lowest at 4.6% for EW (22%) while the adhesiveness of the pasta decreased from −1.26 to −4.37 g.sec with increased concentration of GG but increased (*p* < 0.05) with increased concentrations of EW (−6.82 g.sec to −3.31 g.sec) and XG (−2.85 g.sec to −1.37 g.sec). Unripe banana flour‐based pasta quality parameters can be enhanced using optimal inclusion levels of 19% for EW and 2%–3% for GG and XG.

## Graphical abstract

This research explored the effects of three hydrocolloids, including egg white (EW, 18%–22%), guar gum (GG, 0.5%–4.5%), and xanthan gum (XG, 0.5%–4.5%) on the cooking quality and techno‐functional properties of gluten‐free unripe banana flour pasta. Results showed that increasing levels of the three hydrocolloids improved cooking time, hardness, lightness, and hue, while also affecting cooking loss and adhesiveness. Optimal inclusion levels of the hydrocolloids needed to enhance the quality of pasta were identified as 19% for EW and 2%–3% for GG and XG.

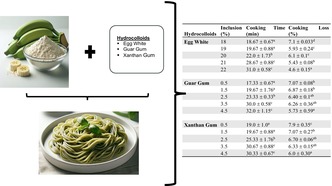

## 1 Introduction

Pasta is among the most consumed foods across the global food systems due to its palatability, long shelf life, minimum cooking, and transportation requirements (Amini Khoozani et al. 2019; Camelo‐Méndez et al. 2018; Woomer and Adedeji 2020). Pasta comprises complex carbohydrates and gluten but lacks dietary fiber, resistant starch, and bioactive compounds (Palavecino et al. 2019). The occurrence of gluten‐related disorders such as celiac disease has negatively impacted the health of consumers who are allergic to gluten. Several studies (Gatti et al. 2024; Kurppa et al. 2024; Burianek et al. 2024) have shown that gluten‐related disorders affect about 1% of the global population, the majority of whom are female; thus, there is a need to develop cost‐effective gluten‐free (GF) products with acceptable sensory properties. Banana flour is a suitable ingredient for the production and manufacturing of GF pasta because it is rich in carbohydrates, vitamins, resistant starch, minerals, and bioactive compounds (Dotto et al. 2019; Tangthanantorn et al. 2021; Dibakoane et al. 2023; Maseko et al. 2024), with some studies suggesting that incorporating banana flour can enhance the quality of GF pasta without significantly altering consumer preferences (Flores‐Silva et al. 2014; Ovando‐Martinez et al. 2009). However, banana flour‐based pasta products exhibit compromised quality properties due to their relatively low protein content and lack of gluten (Rachman et al. 2020; Thakaeng et al. 2021).

Banana flour pasta has been shown to exhibit longer cooking times, high cooking losses, darker color, and poor textural quality, such as stickiness (Padalino et al. 2016; Rachman et al. 2020). Hence, there is a need to incorporate ingredients such as hydrocolloids at optimum levels into banana flour pasta for enhanced quality. Castelo‐Branco et al. (2017) reported that tagliatelle pasta prepared with 15%–30% banana flour and blended with egg protein showed no dark color. Tangthanantorn et al. (2021) demonstrated that incorporating 1.0% and 1.5% guar and xanthan gum significantly reduced the cooking loss of wheat noodles with 30% unripe banana flour. Currently, there are limited studies that have assessed the effect of hydrocolloids on the properties of unmodified unripe banana flour pasta. Yet, hydrocolloids have been shown to improve the properties of proso‐millet pasta (Romero et al. 2017); soya–channa flour pasta (Susanna and Prabhasankar 2013); cassava starch pasta (Milde et al. 2020); red jasmine flour noodles (Kraithong and Rawdkuen 2020); sorghum flour pasta (Palavecino et al. 2017); amaranth flour pasta (Chauhan et al. 2017) and banana flour pasta (Zandonadi et al. 2012). Furthermore, there is a paucity of literature on the combined effect of hydrocolloids such as xanthan gum (XG), guar gum (GG) and egg white (EW) on the cooking time and loss, color, and texture properties of GF unripe banana flour pasta. This work, therefore, seeks to determine the combined effect of graded levels of three hydrocolloids: XG, GG, and EW on the cooking quality, texture, and color of GF unmodified unripe banana flour pasta.

## 2 Materials and Methods

### 2.1 Materials

Dried green banana flour was purchased from a local retail supermarket in Mbombela, South Africa. The composition of banana flour was 4.9% protein, 71% carbohydrates, 0.6% fat, and 12.8% dietary fiber. Protein albumen egg white powder was purchased from Mopani Pharmacy, Mbombela, with a composition of 84% protein, 4.5% carbohydrates, < 0.1% fat, 0% dietary fiber, and 4.5% ash. Guar gum (Essentially Natural, Cape Town) had a composition of 4% protein, 0% carbohydrates, 0.2% fat, and 83% dietary fiber. Xanthan Gum (Dis‐Chem Group, Mbombela) comprised 6% protein, 0% carbohydrates, and fat, and 76% dietary fiber. Salt (Meat World Retail Butchery, Mbombela) comprised 99% sodium chloride.

### 2.2 Pasta Preparation and Formulation

Banana flour samples were formulated according to Zandonadi et al. (2012) with modifications. The pasta with graded levels of a given hydrocolloid was prepared by holding the other hydrocolloids' level constant. All the samples for each hydrocolloid were prepared at five graded levels. Egg white samples were prepared from 18% to 22% (w/w) with both XG and GG held at 2.5% (w/w). Guar gum and XG samples were prepared from 0.5% to 4.5% with EW at 20% with either XG or GG at 2.5%, respectively. In all formulations, salt was maintained at 0.9% while water was added to fill up the decrease from the maximum proportion. The ingredients were mixed manually for 10 min and then with a Kitchen Aid Heavy Duty Bowl‐Lift 4.8 L Stand Mixer (Kitchen Aid, St. Joseph, USA) for 5 min to achieve homogenization. The dough was allowed to rest for 15 min in an air‐tight container. The dough was strained and cut into fettuccine strips using a cylindrical pasta machine (Progressive PL8 Professional Pasta Machine—PL8; Progressive International Corp, China) and then dried overnight at 45°C in a hot‐air oven. The dried pasta samples were stored in air‐tight plastic packs at 25°C ± 2°C till further analysis.

### 2.3 Cooking Quality

The AACC method 66–50 (2000) was used in determining the cooking time and cooking loss of the pasta. For analysis of cooking time, 25 g of banana flour pasta was weighed and cut into 5 cm long pieces. Thereafter, the pasta was transferred into boiling water and stirred to ensure that the pasta pieces separated. The pasta samples were taken from the boiling water and squeezed between two glasses to check whether the core of the pasta had disappeared. The time taken for the white core to disappear was recorded as the cooking time. To determine the cooking loss, the water used to cook the pasta was transferred into a pre‐weighed 500 mL beaker and dried in a hot‐air convection oven for 20 h at 110°C. The weight of the remaining residue (cooking loss) was recorded and expressed as a percentage of the weight of the original pasta sample before cooking using equation 1.

(1) $$\text{Cooking Loss}\;\left(\%\right)=\frac{\text{Remaining Solid Content After Drying}\;\left(g\right)}{\text{Weight of Fresh Pasta}\;\left(g\right)}\;x\;100$$

### 2.4 Color

Pasta samples were cooked according to methods prescribed by the AACC (2000). The samples were rinsed in 100 mL distilled water and rested for 10 min in plastic containers. The color of the cooked pasta was analyzed using a handheld colorimeter (Lovibond Chroma Meter). The color parameters that were analyzed include lightness (L*), redness/greenness (a*), yellowness/blueness (b*), chroma (C*), and the hue angle (H°). The C* and H° were determined using equations 2 and 3 with all color analysis for the pasta samples conducted 10 times.

(2) $$\text{Chroma}\;\left(C*\right)=\sqrt{{\left(a*\right)}^{2}+{\left(b*\right)}^{2}}$$

(3) $$\mathrm{Hue}\;\left(H^{{}^{\circ}}\right)=\tan^{-1}\left\{\frac{b*}{a*}\right\}$$

### 2.5 Texture Analysis

The texture analysis of the cooked pasta samples was done according to methods described by El‐Sohaimy et al. (2020) with modifications. Ten grams of pasta were cooked in 1 L of distilled water containing 2.5 mL of NaCl using cooking times determined in the cooking test. The cooked pasta was rinsed with 100 mL of distilled water and allowed to rest for 10 min at 25°C before it was analyzed for texture. The texture analyzer (TA/TX‐plus; StableMicro system, Surrey, UK) equipped with a 5 kg load cell and the Exponent 32.6.0.2.0 software was used. The texture analyzer was also equipped with a P36 cylindrical probe. The test was done with 2 mm/s for pre‐test, test, and post‐test speeds, a 75% strain, an auto trigger type set at 10 g, and 200 pps (points per second) data acquisition. For each sample, seven (7) replicates were conducted and the hardness (g), gradient modulus (stiffness, g.sec) and adhesiveness (g.sec) determined.

### 2.6 Statistical Analysis

The SAS software V8 (SAS Institute Inc., Cary, NC, USA) was used to analyze obtained data. The influence of the hydrocolloids on the quality of the pasta was analyzed using the One‐way ANOVA. The means between the samples (95% confidence interval) were separated using Duncan's multiple range test. Analyzed properties of cooking time, cooking loss, and texture parameters were replicated seven times, while color properties were conducted in 10 replicates. Response surface regression analysis was carried out to determine the relationships between the inclusion levels of the hydrocolloids and the quality parameters of the pasta. The model used in assessing the relationship between the inclusion levels and the response parameters is shown in equation 4.

(4) $$y={\mathrm{ax}}^{2}+\mathrm{bx}+c$$

where *y* is the response parameter; *a* and *b* are the model coefficients; *c* is the intercept; *x* is the inclusion level of the hydrocolloids; and *‐b/2a* is the *x* value that maximizes or minimizes (optimizes) a response parameter.

## 3 Results and Discussion

### 3.1 Cooking Time

Cooking time refers to the time it takes for the pasta to lose its white core and form a flat, paper‐like position (AACC 2000). The cooking time of pasta increased significantly (*p* > 0.05) in response to incremental levels of each hydrocolloid (Table 1). Similar trends have been reported by Milde et al. (2020), Rachman et al. (2019), and Xie et al. (2020) for cassava starch pasta. The cooking time of the pasta showed a linear increase as the inclusion levels of the hydrocolloids increased, with *R^2^* values ranging from 0.82 to 0.91. This is similar to the findings of Detchewa et al. (2022) who demonstrated that combining EW and soy protein with XG and GG in a single product increased the cooking time of jasmine rice noodles.

**TABLE 1:** Influence of inclusion levels (%) of hydrocolloids on the cooking time and loss of banana flour pasta.

Hydrocolloids	Inclusion (%)	Cooking time (min)	Cooking loss (%)
Egg white	18	18.67 ± 0.67^a^	7.1 ± 0.033^d^
	19	19.67 ± 0.88^a^	5.93 ± 0.24^c^
	20	22.0 ± 1.73^b^	6.1 ± 0.1^c^
	21	28.67 ± 0.88^c^	5.43 ± 0.08^b^
	22	31.0 ± 0.58^c^	4.6 ± 0.15^a^
Guar gum	0.5	17.33 ± 0.67^a^	7.07 ± 0.08^b^
	1.5	19.67 ± 1.76^a^	6.87 ± 0.18^b^
	2.5	23.33 ± 0.33^b^	6.40 ± 0.1^ab^
	3.5	30.0 ± 0.58^c^	6.26 ± 0.36^ab^
	4.5	32.0 ± 1.15^c^	5.73 ± 0.59^a^
Xanthan gum	0.5	19.0 ± 1.0^a^	7.9 ± 0.35^c^
	1.5	19.67 ± 0.88^a^	7.07 ± 0.27^b^
	2.5	25.33 ± 1.76^b^	6.70 ± 0.06^ab^
	3.5	30.67 ± 0.88^c^	6.33 ± 0.15^ab^
	4.5	30.33 ± 0.67^c^	6.0 ± 0.30^a^

*Note:* Values are means of seven replicates ± standard deviation. For each hydrocolloid, means with different superscript letters in each column are significantly different (*p* < 0.05).

The cooking time of pasta fortified with EW (18.67–31 min) was, however, higher than that reported by Detchewa et al. (2022), Sosa et al. (2018) and Xie et al. (2020) for jasmine rice noodles fortified with EW. The cooking time of pasta fortified with GG (17.33–32 min) was within the range reported by Detchewa et al. (2022) for jasmine flour rice noodles (19.22–19.27 min) but higher than those reported by Kraithong and Rawdkuen (2020) for Jasmine rice noodles (8.43–8.53 min). The cooking time of pasta samples fortified with XG was similar to those reported by Detchewa et al. (2022) of 22.40–25.20 min for jasmine rice flour noodles but significantly higher than those reported by Kraithong and Rawdkuen (2020) for jasmine rice flour noodles, Martín‐Esparza et al. (2018) for tiger nut flour pasta, and Milde et al. (2020) (2.0–8.2 min) for cassava starch pasta. The higher cooking time associated with increasing inclusion levels of EW, XG, and GG could be attributed to the formation of a compact pasta structure, which retards the penetration of water into the pasta core (Zandonadi et al. 2012; Xie et al. 2020; Detchewa et al. 2022; Sosa et al. 2018), thus requiring more time and higher temperatures to gelatinize the starch granules.

### 3.2 Cooking Loss

Cooking loss refers to the number of solids that leach out of the pasta structure during cooking (Sosa et al. 2018; Zandonadi et al. 2012). The recommended/acceptable cooking loss values for pasta samples range between 8% and 10%. The cooking loss of the pasta significantly (*p* < 0.05) decreased in response to the incremental levels of the hydrocolloids (Table 1). Cooking loss showed a linear decrease (*R^2^* = 0.44–0.85) in response to the incremental levels of the hydrocolloids. Cooking loss of pasta fortified with EW (4.6%–7.1%) was within the range reported by Marti et al. (2013) and Rachman et al. (2019) for banana flour and rice pasta (4.71%–9.71%). The low cooking loss of fortified pasta was probably due to the formation of a more stable and extensive structure. Zheng et al. (2016) demonstrated that banana flour and semolina pasta samples fortified with EW displayed a more extensive and strong protein structure compared to those fortified only with GG and XG. Marti et al. (2013) further reported that egg albumen was more effective in reducing the cooking loss of rice flour pasta compared to whey proteins.

The cooking loss reported in the present study for GG‐fortified pasta samples (5.73%–7.07%) was higher than those reported by Kraithong and Rawdkuen (2020) and Tangthanantorn et al. (2021) for extruded jasmine rice noodles (5.27%–5.32%) and dried banana flour samples (3%–4.02%). Guar gum reduces the cooking loss of banana pasta samples by interacting with the starch granules through hydrogen and hydrophilic interactions, which encapsulates the starch granules and prevent amylose diffusion during cooking (Tangthanantorn et al. 2021). The findings reported in the present study for XG‐infused unripe banana flour pasta (6%–7.9%) were similar to those reported by Milde et al. (2020) and Kraithong and Rawdkuen (2020) for cassava starch‐corn flour pasta samples (5.4%–8.4%) and extruded jasmine flour noodles (7.25%–7.97%). Kraithong and Rawdkuen (2020) suggested that the highly branched nature of XG increased the cooking loss of extruded rice noodles. A highly branched molecule is characterized by high rehydration, which may soften and weaken the pasta structure and trigger the diffusion of solids during cooking (Kraithong and Rawdkuen 2020; Tangthanantorn et al. 2021).

### 3.3 Texture Analysis

#### 3.3.1 Hardness

Gluten‐free foods such as pasta are sticky and sometimes undesirable due to the lack of a stable protein matrix and the inability of GF flour to withstand overcooking or high temperatures (Rachman et al. 2020; Rachman et al. 2019; Padalino et al. 2013). The incorporation of hydrocolloids improves the texture of gluten‐free foods by interacting with starch granules and by reducing the loss of solids from the pasta structure (Tangthanantorn et al. 2021; Xie et al. 2020; Zheng et al. 2016; Guo et al. 2020; Widelska et al. 2019). This increases the water absorption capacity of the pasta by forming a protective layer around the starch granules, thus improving their resilience during the cooking process (Gasparre and Rosell 2019). In the present study, the hardness of the pasta fortified with hydrocolloids increased with an increase in the concentration of the hydrocolloids. The adhesiveness (except for pasta fortified with EW and XG) and gradient modulus (stiffness) decreased in response to the incremental levels of the hydrocolloids (Table 2). Similar trends were observed by Larrosa et al. (2016) for corn‐based pasta, Xie et al. (2020) for rigatoni pasta, Tangthanantorn et al. (2021) for banana flour pasta, and Rafiq et al. (2016) for horse chestnut flour noodles.

**TABLE 2:** Influence of inclusion levels (%) of hydrocolloids on the textural properties of banana flour pasta.

Hydrocolloids	Inclusion (%)	Textural properties		
		Hardness (g)	Adhesiveness (g.sec)	Gradient modulus (g.sec)
Egg white	18	4373.99 ± 164.07^a^	−6.82 ± 1.20^a^	6397.97 ± 182.52^a^
	19	4832.82 ± 60.64^ab^	−4.79 ± 1.04^ab^	7156.83 ± 203.46^b^
	20	4885.48 ± 231.72^b^	−3.99 ± 0.38^ab^	6792.33 ± 145.40^ab^
	21	5232.32 ± 66.88^bc^	−3.69 ± 0.26^b^	6713.73 ± 140.62^ab^
	22	5394.13 ± 132.07^c^	−3.31 ± 1.26^b^	6525.82 ± 150.15^a^
Guar gum	0.5	4207.72 ± 152.34^a^	−1.26 ± 0.53^b^	6894.79 ± 238.28^ab^
	1.5	5012.32 ± 209.25^b^	−3.18 ± 1.12^ab^	7608.86 ± 284.49^c^
	2.5	5229.17 ± 61.41^b^	−4.22 ± 0.51^a^	7281.15 ± 157.24^bc^
	3.5	5380.85 ± 108.61^b^	−4.19 ± 0.27^a^	6919.91 ± 126.67^ab^
	4.5	5274.54 ± 104.27^b^	−4.37 ± 0.61^a^	6470.28 ± 27.52^a^
Xanthan gum	0.5	4785.50 ± 200.45^a^	−1.98 ± 0.62^a^	7506.71 ± 402.30^b^
	1.5	5042.44 ± 102.22^a^	−2.85 ± 1.05^a^	7631.23 ± 130.98^b^
	2.5	5206.16 ± 214.13^a^	−1.45 ± 0.35^a^	7229.49 ± 216.75^b^
	3.5	4893.72 ± 257.44^a^	−1.37 ± 0.83^a^	6357.76 ± 251.52^a^
	4.5	5318.58 ± 162.15^a^	−1.71 ± 0.55^a^	6003.86 ± 98.47^a^

*Note:* Values are means of seven replicates ± standard deviation. For each hydrocolloid, means with a different superscript in each column are significantly different (*p* < 0.05).

Hardness measures the force required to compress food between the tongue and palate of any penetration (Park et al. 2020). Food products with high hardness (firmness) are deemed high quality due to enhanced textural properties (Park et al. 2020). The hardness of the pasta increased from 4373.99 g to 5394.13 g in response to incremental levels of EW. In the present study, the hardness of the pasta fortified with 22% EW was significantly higher than that fortified with 18%–21% EW. This trend has been reported by Bai et al. (2022) and Guo et al. (2020) who reported that the hardness of the pasta increased in response to incremental levels of EW. The hardness values reported in the present study are higher than those reported for corn flour‐starch pasta (612.85 g–3485.39 g) (Larossa et al. 2016) and rigatoni pasta (132.56 g–244.73 g) (Xie et al. 2020). These variations could have been due to the use of relatively low inclusion levels of EW in the reported studies compared to those used in the present study, especially as the hardness of pasta depends on the inclusion levels of the hydrocolloids Witek et al. (2020).

The hardness of the pasta fortified with GG increased in response to incremental levels of GG. The hardness of pasta fortified with 1.5%–4.5% GG was significantly higher (*p* < 0.05) than that fortified with 0.5% GG. The hardness of pasta fortified with GG was lower than that of tiger nut flour pasta (10986 g–12,765 g) (Gasparre and Rosell 2019) but higher than those of extruded rice flour pasta (1766.10 g–1798.58 g) (Kraithong and Rawdkuen 2020) and proso‐millet pasta (4364.39 g–4568.33 g) (Romero et al. 2017). The increase in the hardness values of the pasta is due to the ability of GG to interact with the starch granules to form a stable matrix inside the pasta (Gasparre and Rosell 2019).

The hardness of the pasta fortified with XG increased in response to the incremental levels of XG. The hardness of the pasta fortified with 4.5% XG was higher than that fortified with 0.5%–3.5% XG, with no significant difference (*p* > 0.05) in the hardness of the pasta fortified with XG. The hardness values of pasta fortified with XG are higher than those of non‐fried potato noodles (20.9 g–3690.12 g) (Javaid et al. 2021), rice flour pasta (807.61 g) (Sanguinetti et al. 2015) and proso‐millet flour pasta (4364.39 g–4568.33 g) (Romero et al. 2017), but significantly lower than that of tiger nut flour pasta (10349 g–10,711 g) (Gasparre and Rosell 2019). The presence of ionic charges enables XG to increase the hardness of the pasta by interacting and binding soluble starch granules to enhance the structural compactness of the pasta (Milde et al. 2020; Widelska et al. 2019).

#### 3.3.2 Adhesiveness

The hardness of the pasta has been shown to reduce the adhesiveness of the pasta, which might be due to the restricted amylose diffusion (Guo et al. 2020). Adhesiveness is the adherence of leached or diffused starch granules on the surface of pasta (Guo et al. 2020). It is also described as the force required to remove food that is adhered to the mouth (Park et al. 2020). High adhesiveness is not desirable, as it implies that the pasta has a sticky texture, an undesirable property to consumers (Padalino et al. 2013). The adhesiveness of pasta fortified with GG decreased in response to the incremental levels of GG. Similar trends have been reported for extruded rice flour pasta (Kraithong and Rawdkuen 2020) and whole amaranth flour pasta (Chauhan et al. 2017). The adhesiveness values of the pasta fortified with GG (−1.26 g.sec to −4.37 g.sec) were higher than those of buckwheat noodles (−24.31 g.mm to −84.60 g.mm) (Jang et al. 2015); whole amaranth flour pasta (−16.52 g.sec–36.31 g.sec) (Chauhan et al. 2017) but lower than that of rice flour pasta (305.91 g.sec–414 g.sec) (Sanguinetti et al. 2015). The decrease in the adhesiveness due to the ability of GG to form a stable protein matrix prevents excess leaching of solids from the pasta during cooking, thereby resulting in less sticky pasta (Tangthanantorn et al. 2021; Gasparre and Rosell 2019). However, the adhesiveness of pasta fortified with EW and XG increased in response to incremental levels of EW and XG. The adhesiveness of the pasta showed a linear decrease (*R^2^* = 0.4007; *p* = 0.0112) in response to incremental levels of EW. The findings of the present study are similar to those reported for corn‐based pasta made with 0.25 and 0.42% EW and whose adhesiveness values increased from 1.41% to 1.82% and 1.12% to 1.22% as the moisture content in the pasta formulation increased 36.15%–37.5% (Larossa et al. 2016). It is suggested that high moisture and less protein content increase the adhesiveness of the pasta due to the lack of a compact structure. Findings from this study contradicts that of Guo et al. (2020) who demonstrated that increasing egg white levels in the pasta formulation reduced the adhesiveness of oat noodles. It also contradicted the hypothesis that aggregation of proteins enables pasta to withstand cooking and have less stickiness Guo et al. (2020). The increase in the adhesiveness of the pasta fortified with EW may be attributed to prolonged cooking times, which may have caused solids to escape the pasta matrix (Xie et al. 2020). Numerically, the adhesiveness values of the pasta fortified with EW are lower than those reported for corn‐based pasta (67.30–158.89 g.sec) (Larossa et al. 2016) and oat noodles (82.60–138.88 g.mm) (Guo et al. 2020).

The high‐water absorption capacity of the XG causes the pasta to have more stickiness (Gasparre and Rosell 2019; Thuy et al. 2023). Xanthan gum cannot form a more robust protein matrix like GG and EW; hence, some studies concluded that pasta fortified with GG had more firmness compared to XG (Gasparre and Rosell 2019; Sanguinetti et al. 2015). The adhesiveness values of the XG fortified pasta are lower than those of horse chestnut flour noodles (30.59 g/mm–50.99 g/mm) (Rafiq et al. 2016), tiger nut flour noodles (70 g.sec–1144 g.sec) (Gasparre and Rosell 2019), and cassava starch noodles (20.39 g.sec) (Milde et al. 2020), but higher than that reported for spinach noodles (−30 g.sec–90 g.sec) (Shere et al. 2020) and extruded flour noodles (−40.70 g.sec–41.27 g.sec) (Kraithong and Rawdkuen 2020).

#### 3.3.3 Gradient Modulus

The gradient modulus is used in material physics to measure a given material's elasticity or response to elastic or springy deformation (Askadskii et al. 2017; Jones and Ashby 2018). Materials with a high gradient modulus stretch very little when pulled, while those with low modulus materials stretch a lot (Jones and Ashby 2018). It has been suggested that incorporating hydrocolloids improves the elasticity of pasta (Tangthanantorn et al. 2021; Xie et al. 2020). In the present study, the gradient modulus of the pasta decreased (*p* < 0.05) in response to incremental levels of each hydrocolloid (Table 2). These findings are similar to those of previous studies that showed that incorporating EW, XG, and GG increased the elasticity of rigatoni pasta (Xie et al. 2020) and extruded rice flour noodles (Kraithong and Rawdkuen 2020). This demonstrated that these hydrocolloids could interact with molecules such as starch granules in banana flour to enhance the elasticity of the pasta dough and banana flour pasta.

### 3.4 Color

The lightness of pasta fortified with 21% EW was greater than that fortified with 18%–19% EW (Table 3). The L* values reported for pasta fortified with EW are significantly lower than those reported for extruded jasmine flour noodles (67.87–69.90) (Detchewa et al. 2022) and white‐black sorghum flour pasta (47.27–65.17) (Palavecino et al. 2017). These variations could be due to differences in the lightness of the ingredients used in the reported studies. The L* of the pasta showed linear responses (*R^2^* = 0.1790; *p* < 0.001) in response to incremental levels of EW. The L* of the pasta fortified with GG increased in response to incremental levels of GG. Furthermore, the L* of pasta fortified with 4.5% GG was significantly greater (*p* < 0.05) than that of pasta fortified with 0.5%–3.5% GG. The L* values of pasta fortified with GG are higher than those reported for soy‐channa flour pasta (31.16) but significantly lower than those reported for rice starch milk pasta (71.17–72.49) (Sanguinetti et al. 2015) and extruded jasmine rice flour noodles (67.21–66.90) (Detchewa et al. 2022). These variations could be due to the use of relatively lighter ingredients in the previous studies compared to the banana flour used in this study. Furthermore, the L* of the pasta fortified with XG increased as the inclusion levels of XG increased. The lightness of pasta fortified with 3.5% XG was significantly greater (*p* < 0.05) than that of pasta fortified with 0.5%–2.5% XG and 4.5% XG. The L* values reported for pasta fortified with XG are lower than those reported for proso‐millet pasta (76.87–77.27) (Romero et al. 2017), black sorghum flour pasta (47.27–57.17) (Palavecino et al. 2017) and rice pasta with autoclaved starch (45.3–54.48) (Raungrusmee et al. 2020). The increase in lightness may be attributed to the ability of hydrocolloids to form a formation of a protective layer around starch granules, which prevents excess leaching and preserves the lightness of the pasta (Shahzad et al. 2019). Egg white preserves and increases the lightness of pasta by enhancing the bonding of starch granules and proteins to form a stable protein matrix that prevents excess solids from the pasta structure (Xie et al. 2020; Sosa et al. 2018; Zheng et al. 2016; Detchewa et al. 2022; Saha and Bhattacharya 2010). Gums also prevent the discoloration of pasta by interacting with amylopectin to increase the viscosity and reduce excess loss of solids (Raungrusmee et al. 2020). The increase in the lightness of the cooked pasta could also be due to starch gelatinization, which causes the lightness of pasta to increase (Sanguinetti et al. 2015).

**TABLE 3:** Influence of the inclusion levels of hydrocolloids on the color properties of banana flour‐based pasta.

Hydrocolloids	Inclusion (%)	*L*	*a*	*b*	C	H°
Egg white	18	36.3 ± 0.40^a^	7.17 ± 0.122^b^	11.5 ± 0.21^b^	13.6 ± 0.23^bc^	58.0 ± 0.32^b^
	19	36.3 ± 0.21^a^	7.26 ± 0.086^b^	12.5 ± 0.15^c^	14.4 ± 0.16^d^	59.6 ± 0.28^c^
	20	37.1 ± 0.15^b^	7.19 ± 0.059^b^	11.9 ± 0.12^b^	13.9 ± 0.13^cd^	58.7 ± 0.30^bc^
	21	37.9 ± 0.15^c^	6.88 ± 0.098^a^	11.6 ± 0.16^b^	13.4 ± 0.17^b^	59.2 ± 0.26^c^
	22	37.8 ± 0.20^bc^	6.90 ± 0.080^a^	10.5 ± 0.17^a^	12.6 ± 0.17^a^	56.7 ± 0.35^a^
Guar gum	0.5	37.0 ± 0.29^a^	7.38 ± 0.123^b^	12.8 ± 0.15^c^	14.9 ± 0.18^d^	60.1 ± 0.32^b^
	1.5	37.6 ± 0.39^ab^	7.07 ± 0.115^ab^	12.4 ± 0.13^c^	14.3 ± 0.14^c^	60.1 ± 0.50^b^
	2.5	37.5 ± 0.29^ab^	7.27 ± 0.142^b^	12.4 ± 0.18^c^	14.4 ± 0.19^cd^	59.8 ± 0.49^b^
	3.5	38.1 ± 0.21^bc^	7.09 ± 0.085^ab^	11.9 ± 0.18^b^	13.8 ± 0.18^b^	59.0 ± 0.30^ab^
	4.5	38.6 ± 0.16^c^	6.90 ± 0.101^a^	11.2 ± 0.15^a^	13.1 ± 0.17^a^	58.3 ± 0.28^a^
Xanthan gum	0.5	33.2 ± 0.20^a^	7.72 ± 0.068^c^	12.5 ± 0.16^b^	14.7 ± 0.16^bc^	58.2 ± 0.29^a^
	1.5	36.0 ± 0.32^b^	7.68 ± 0.112^c^	12.9 ± 0.17^c^	15.1 ± 0.19^c^	59.3 ± 0.27^b^
	2.5	37.5 ± 0.33^bc^	7.22 ± 0.078^b^	12.4 ± 0.15^b^	14.3 ± 0.16^b^	59.7 ± 0.25^b^
	3.5	40.2 ± 0.14^d^	6.83 ± 0.075^a^	11.6 ± 0.16^a^	13.5 ± 0.16^a^	59.3 ± 0.42^b^
	4.5	38.9 ± 1.21^cd^	6.65 ± 0.102^a^	11.4 ± 0.12^a^	13.3 ± 0.16^a^	59.9 ± 0.26^b^

*Note:* Values are means of 10 replicates ± standard deviation. For each hydrocolloid, means with a different superscript in each column are significantly different (*p* < 0.05).

The redness (*a*) of pasta samples fortified with 18%–20% EW was significantly greater (*p* < 0.05) than that fortified with 21%–22% EW. The *a* values of pasta fortified with EW are higher than those reported for the jasmine rice flour noodles (0.15–0.17) (Detchewa et al. 2022) but lower than those reported for black sorghum flour pasta (7.87–10.87) (Palavecino et al. 2017). The decrease in redness of the pasta is probably due to the thermal degradation of the carotenoids present in the egg white due to their heat‐liable nature (Romero et al. 2017). Furthermore, pasta fortified with 4.5% GG showed significantly lower (*p* < 0.05) redness than that of pasta fortified with 0.5%–3.5% GG. The redness values reported in this study were higher than those of soya‐channa flour pasta (4.77) (Susanna and Prabhasankar 2013), jasmine rice noodles (0.17–0.21) (Detchewa et al. 2022), and rice flour‐corn‐starch pasta (−1.89 to −2.03) (Sanguinetti et al. 2015). Likewise, pasta fortified with 3.5% and 4.5% XG showed significantly lower (*p* < 0.05) brightness compared to pasta fortified with 0.5%–2.5% XG. The a* value of the pasta fortified with XG is higher than that reported by Milde et al. (2020) for cassava starch noodles (1–2), black sorghum flour pasta (7.48–10.87) (Palavecino et al. 2017), and rice flour pasta (−1.89 to ‒3.59) (Sanguinetti et al. 2015). The higher *a* values obtained in this study, which compare to those in the literature, could be due to the use of relatively lighter ingredients such as banana flour. It could also be due to the interaction of the gums and carotenoids in banana flour, which improve the brightness and redness of the pasta, given that redness is positively associated with brightness (Kraithong and Rawdkuen 2020; Javaid et al. 2021). It has been shown that pigments that influence the redness of pasta are heat‐liable and degrade when subjected to high temperatures (Aravind et al. 2012; Motta Romero et al. 2017). Hence, it can be suggested that the decreasing *a* values in response to incremental levels of hydrocolloids in the present study could be due to the longer cooking time, which exposes the pigments to higher temperatures for prolonged periods, thereby increasing their degradation.

The *b* values of the cooked pasta decreased in response to the incremental levels of the hydrocolloids (Table 3). The yellowness of pasta fortified with 22% EW was significantly lower (*p* < 0.05) than that of pasta fortified with 18%–21% EW. The *b* values of pasta fortified with EW are comparable to those reported for extruded jasmine rice flour noodles (Detchewa et al. 2022) but lower than those reported for white sorghum flour pasta (17.33) (Palavecino et al. 2017). The *b* values of pasta fortified with EW showed a quadratic response of *R^2^* = 0.1753 (*p* < 0.0001) in response to the incremental levels of EW. Furthermore, pasta fortified with 0.5%–2.5% GG had significantly higher (*p* < 0.05) *b* values compared to pasta fortified with 3.5%–4.5% GG. The obtained b* values of pasta fortified with GG are comparable to those of soya‐channa flour pasta (10.64) (Amini Khoozani et al. 2019) and sorghum flour pasta (12.38–17.33) (Camelo‐Méndez et al. 2018) but are lower than those reported for cassava starch pasta (22–28) (Woomer and Adedeji 2020). Furthermore, pasta fortified with 0.5%–2.5% XG had significantly higher (*p* < 0.05) *b* values compared to pasta fortified with 3.5%–4.5% XG. The *b* values of pasta fortified with XG are higher than those reported for rice starch–milk protein pasta (8.66–8.55) (Sanguinetti et al. 2015) and comparable to those reported for rice‐autoclaved‐starch pasta (14.22–16.35) (Raungrusmee et al. 2020) and proso‐millet flour pasta (15.84–16.04) (Motta Romero et al. 2017). In this study, the *b* values of the pasta decreased in response to increasing inclusion levels of the hydrocolloids. This trend may be attributed to the destruction of pigments in the ingredients as the pasta undergoes thermal processing (Motta Romero et al. 2017). Aravind et al. (2012) reported that the redness and yellowness of both cooked pastas decreased substantially at 10%–20% GG. The low *b* values of pasta fortified with EW could be due to the destruction of carotenoids, which results in less reflected light (Bai et al. 2022; Lorenzo et al. 2018).

Pasta fortified with 19% EW had a significantly higher (*p* < 0.05) mean saturation index (C) compared to pasta fortified with 18% and 20%–22% EW. Furthermore, the C value of pasta fortified with 0.5% GG was significantly higher than those fortified with 1.5%–4.5% GG. The C values of pasta fortified with 1.5% XG were significantly greater (*p* < 0.05) than those reported for pasta fortified with 0.5% XG and 2.5%–4.5% XG. Pasta fortified with 4.5% GG and XG had the lowest C values compared to pasta with 0.5%–3.5% GG and XG. However, the C values of the cooked pasta decreased in response to incremental levels of the hydrocolloids. The decreasing C values of pasta with increasing inclusion levels of the hydrocolloids could be due to the thermal degradation of carotenoids in EW and pigments of banana flour and hydrocolloids caused by prolonged cooking times and heat.

The H° of pasta fortified with 0.5% XG was significantly lower (*p* < 0.05) than that of pasta fortified with 1.5%–4.5% XG, with those fortified with 4.5% XG having the highest value (59.88). Xanthan gum increased the H° of the pasta by preventing excess loss of solids, which preserves the color of the pasta and causes more reflection of light. However, the pasta fortified with EW and GG had lower H° values at higher inclusion levels. Moreover, pasta fortified with 4.5% GG had a significantly lower (*p* < 0.05) compared to pasta fortified with 0.5%–3.5% GG. The low H° value of the pasta fortified with EW and GG at high inclusion levels could be due to the thermal degradation of pigments present in the banana flour (Romero et al. 2017) which results in less reflected light. Sanguinetti et al. (2015) and Kraithong and Rawdkuen (2020) suggested that the inherent properties of the ingredients used in manufacturing the pasta confer more influence on the pasta's color than the hydrocolloids. Hence, Palavecino et al. (2017) found that the lightness of white sorghum pasta was significantly greater than that of black sorghum flour pasta, even though both pasta types were fortified with egg white and egg protein. It can thus be recommended that the positive effects of hydrocolloids on the color of pasta products can be enhanced by compositing them with higher‐lightness ingredients.

## 4 Conclusion

This work demonstrated that the inclusion of hydrocolloids at varying levels in unmodified banana flour can enhance the quality of banana flour pasta through reduced cooking loss as well as increased lightness and hardness. The EW, GG, and XG hydrocolloids used in this study were shown to interact with starch granules in the banana flour to form a more stable and compact protein matrix that reduced water penetration and loss of solids from the pasta structure. This demonstrates that hydrocolloids can increase the acceptability and popularity of banana flour pasta in the general population, thereby contributing positively to human health. Furthermore, the ability of the hydrocolloids to modulate the textural properties, enhance water holding capacity, thicken the batter, and stabilize the structure of gluten‐free flours through interactions with starch granules implies that banana flour can be used in producing bakery products. Outcomes from this work revealed that to enhance the cooking time, color, hardness, adhesiveness, and gradient modulus of banana flour pasta, inclusion levels of 19%–20% for EW and 2%–3% for GG and XG are recommended. The potential cost implications of using the different hydrocolloids as well as consumer acceptability of the banana flour pasta were not conducted in this work; hence, further research is suggested in this regard.

## Author Contributions

**Siphosethu R. Dibakoane:** conceptualization (equal); data curation (equal); formal analysis (lead); investigation (lead); methodology (equal); writing – original draft (lead); writing – review and editing (equal). **Victor Mlambo:** conceptualization (supporting); formal analysis (supporting); investigation (equal); methodology (equal); supervision (equal); writing – review and editing (equal). **Belinda Meiring:** conceptualization (supporting); formal analysis (supporting); investigation (supporting); validation (equal); writing – review and editing (equal). **July Johannes Sibanyoni:** conceptualization (supporting); investigation (supporting); validation (equal); writing – review and editing (equal). **Tonna A. Anyasi:** conceptualization (supporting); investigation (equal); methodology (equal); supervision (equal); writing – original draft (supporting); writing – review and editing (equal). **Obiro Cuthbert Wokadala:** conceptualization (lead); data curation (equal); formal analysis (equal); methodology (supporting); funding acquisition (lead); supervision (lead); validation (equal); writing – original draft (supporting); writing – review and editing (equal).

## Conflicts of Interest

The authors declare no conflicts of interest.

## Data Availability

Data will be made available upon reasonable request.
